# Protective effects of saponin on a hypertension target organ in spontaneously hypertensive rats

**DOI:** 10.3892/etm.2012.856

**Published:** 2012-12-11

**Authors:** MING CHEN, ZIJIANG LONG, YAJUAN WANG, JINLIN LIU, HAI PIAN, LIANG WANG, ZHIWU CHEN

**Affiliations:** 1 Department of Pharmacology, Integrative Medicine Clinical College, Anhui University of Traditional Chinese Medicine; Hefei, Anhui 230032;; 2 Department of Pharmacology, Basic Medical College, Anhui Medical University, Hefei, Anhui 230032;; 3 Department of Pharmacology, Anqing Medicine College, Anqing, Anhui 246052, P.R. China

**Keywords:** hypertension, renin-angiotensin-aldosterone system, saponin

## Abstract

The present study was undertaken to investigate the protective effects of saponin on a hypertensive target organ (the kidney) in spontaneously hypertensive rats (SHRs) and also to explore the effect of saponin on the renin-angiotensin-aldosterone system (RAAS). A total of 24, 14-week-old SHRs were randomly divided into three groups; the first was administered low-dose saponin, the second with high-dose saponin and the third with a placebo as the control group. An additional eight healthy male Wistar rats were used as the normal group. The blood pressures (BPs) of the rats were determined using an animal BP-6 non-invasive blood pressure tester. Furthermore, the gene expression of TGFB1, collagen I and prorenin receptor (PRR) was determined by quantitative real time (qRT)-PCR. The histopathological and morphological features of the tissue samples were assessed semi-quantitatively. The content of saponin in the renal samples was lower in SHRs than in the normal healthy rats, but the plasma levels of saponin were similar. Mean arterial pressure (MAP) was reduced 5 days subsequent to saponin treatment by 36±3 and 51±4 mmHg in the low- and high-dose saponin groups, respectively. The anti-hypertensive effect of saponin was dose-related during the first 4 weeks of treatment. The gene expression of TGFB1 and collagen I in the renal samples was significantly suppressed in the low- and high-dose saponin groups compared with that in the control group. The gene expression of PRR was significantly and dose-dependently increased in the saponin-treated groups. These findings suggested that saponin reduced systemic BP and blocked the circulating and tissue RAAS.

## Introduction

The renin-angiotensin-aldosterone system (RAAS) is a key regulator of blood pressure (BP) and body fluid volume, acting primarily via the effects of angiotensin II (Ang II). The RAAS may increase the load on the cardiovascular system when activated by electrolyte abnormalities, wall stress, pressure and volume (1). Classically, there is an increased release of renin from the granular cells and in turn, an increased conversion of angiotensinogen to angiotensin I from the liver. Angiotensin I is converted to Ang II by the angiotensin-converting enzyme (ACE). Increased Ang II ultimately results in an increased adrenal aldosterone release. Ang II and aldosterone then have various effects on their target organs. In addition to this traditional angiotensin production method, RAAS-independent local angiotensin production has also been described (2), as well as ACE-independent production pathways for the formation of Ang II (3,4).

The RALES trial showed that the maximum benefit of spironolactone was achieved in congestive heart failure patients with the increased levels of collagen synthesis markers (5). Neurohumoral, genetic and mechanical para meters affect the operation of this conversion process and in turn are positively affected by the inhibition of the RAAS-ACE inhibitors and Ang II type 1 (AT1) receptor antagonists, preventing the effects of Ang II, but not the negative effects of aldosterone. Even following the complete inhibition of the RAAS by the ACE inhibitors (6,7) and the administration of additional AT1-receptor antagonists (8), elevated aldosterone levels remain evident in heart failure, indicating the potential gain that may occur from an additional inhibitor of aldosterone.

The aim of the present study was to investigate the protective effects of saponin on a hypertensive target organ (the kidney) in spontaneously hypertensive rats SHRs and also to explore the effects of saponin on the RAAS.

## Materials and methods

### Animals

A total of 24 male or female, 14-week-old SHRs weighing 200–250 g were used in the present study. The SHRs were provided by the Beijing Vital River Laboratory Animal Technology Co., Ltd. (Beijing) with Animal Production License No. SCXK 2007-001. Animals were kept in cages, with controlled light/dark cycles and temperatures, fed with a normal rat chow and had free access to tap water. The SHRs were randomly divided into three groups; the first was adminstered low-dose saponin (27 mg/kg, n=8), the second with high-dose saponin (108 mg/kg, n=8) and the third with a placebo as the control group (n=8). Another eight healthy male Wistar rats were used as the normal group.

### BP measurements

The BPs of the rats were determined using an animal BP-6 non-invasive blood pressure tester after 0, 4, 8, 12 and 10 weeks of drug intervention.

### In situ hybridization

Total mRNA was isolated from the kidney cells of male and female rats at 14 weeks of age. An Ribonuclease Protection Assay (RPA) was performed according to the manufacturer’s instructions (Ambion RPA II kit, Foster City, CA, USA). For each hybridi zation reaction, 40 pg RNA and 50,000 cpm of ^32^P-labeled transcripts were purified at 42°C overnight. Correct expression of the transgene was studied by *in situ* hybridization, as described above. In short, a ^35^S-UTP-labeled mRNA probe was built using a fragment of 600 bp.

### Quantitative real-time (qRT)-PCR

The purification of total RNA from the cells was performed using the NucleoSpin RNA kit I (Macherey-Nagel, Düren, Germany), according to the manufacturer’s instructions. cDNA corresponding to 50 ng of RNA was added to the SYBR-Green JumpStart Taq Ready Mix (Sigma-Aldrich, St. Louis, MO, USA). Following this addition, the cDNA for the 18S rRNA gene was diluted due to the quantitative superiority of the ribosomal RNA. A duplicate was made for each sample. qRT-PCR (Mx4000, Stratagene, TX, USA) was performed using a three-step protocol, followed by a melting curve analysis to verify the homogeneity of the amplified PCR products.

### Statistical analysis

The data are presented as the mean ± SD. Comparisons between the groups of data were performed by using a Student’s t-test. P<0.05 was considered to indicate a statistically significant difference. Data were analyzed with the SPSS 18.0 statistical software package (SPSS Inc., Chicago, IL, USA).

## Results

### Renal distribution of saponin

**Figure 1.**
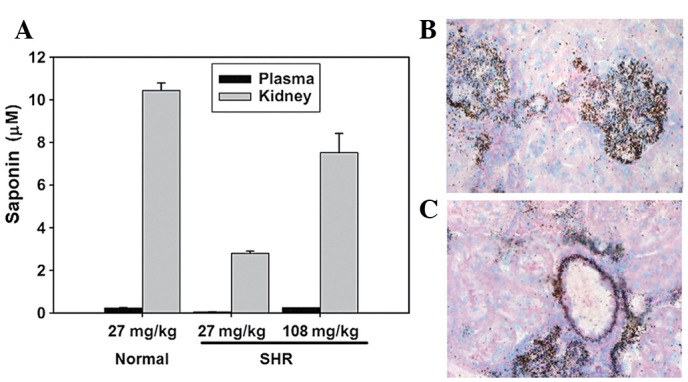
Renal distribution of saponin. (A) Saponin levels in the plasma and kidneys of normal rats and SHRs. (B) Presence of saponin in the glomeruli. (C) Presence of saponin in the vascular wall. SHRs, spontaneously hypertensive rats.

Following treatment with the saponin compound for 2 weeks the plasma saponin levels were 127±18 ng/ml in normal rats (27 mg/kg per day) and 29.7±5.6 and 129±23.7 ng/ml in the SHRs (27 and 108 mg/kg per day, respectively). The mean kidney:plasma concentration ratio of saponin in rats treated with the compound for 2 weeks was 45.6 for the normal rats (27 mg/kg per day) and 30.7 and 63.6 for the SHRs (27 and 108 mg/kg per day, respectively; Fig. 1A), indicating extensive saponin levels in the kidneys. In the rats treated with 27 mg/kg saponin per day, the renal and plasma saponin levels were lower in the SHRs compared with those in the normal rats.

Using light microscopy, autoradiographic grains were observed in the glomeruli of each renal section and used to indicate the presence of saponin (Fig. 1B). Extensive saponin levels were also located in the arterial wall of the small cortical vessels in the kidney (Fig. 1C).

### BP declines in saponin-treated SHRs

**Figure 2.**
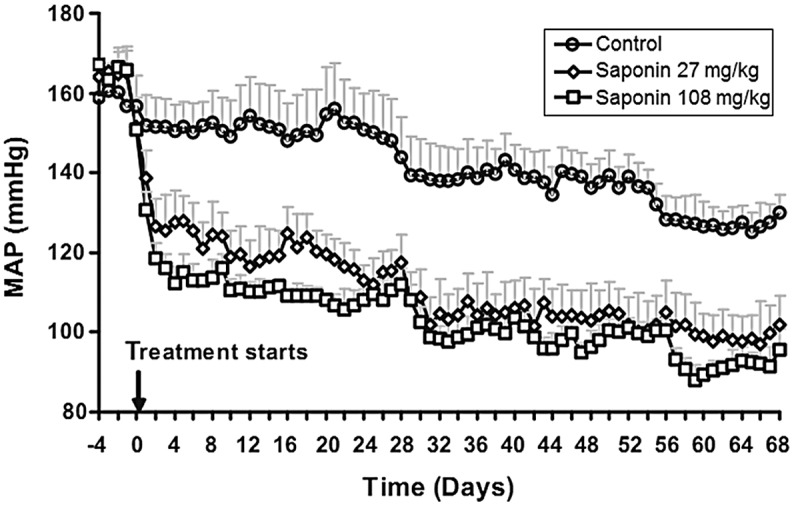
Blood pressure following the saponin treatment. P<0.05 for control vs. saponin groups. MAP, mean arterial pressure.

There was a trend towards a mild and gradual decline in BP in the saponin-treated SHRs (Fig. 2). By contrast, initiation of the saponin treatment caused a prompt and sustained reduction in mean arterial pressure (MAP). MAP was decreased by 36±3 and 51±4 mm Hg in the low- and high-dose groups, respectively, 5 days after the saponin treatment. No significant differences were observed in heart rates following saponin treatment (data not shown).

### Treatment with saponin suppresses gene expression of TGFB1 and collagen I

**Figure 3.**
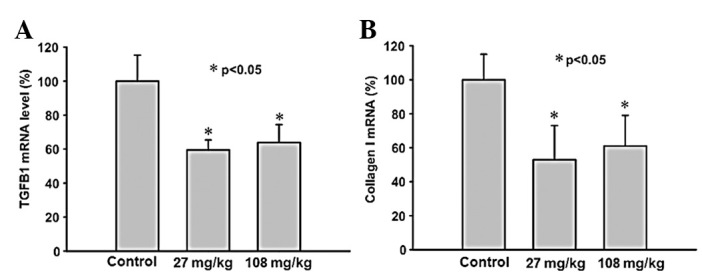
Saponin treatment suppresses the gene expression of (A) TGFB1 and (B) collagen I in renal samples of spontaneously hypertensive rats.

The TGFB1 gene expression in the renal samples was significantly suppressed in the saponin-treated SHRs (Fig. 3A) compared with the controls, with the expression levels in the 27 mg/kg per day group tending to be slightly, but not significantly, more supressed than the 108 mg/kg per day group. The gene expression of collagen I in the renal samples was significantly reduced in each of the saponin-treated SHRs groups (Fig. 3B). No significant differences were observed in the expression of collagens III and V between the saponin-treated SHRs and the controls (data not shown).

### Treatment with saponin suppresses gene expression of PRR

**Figure 4.**
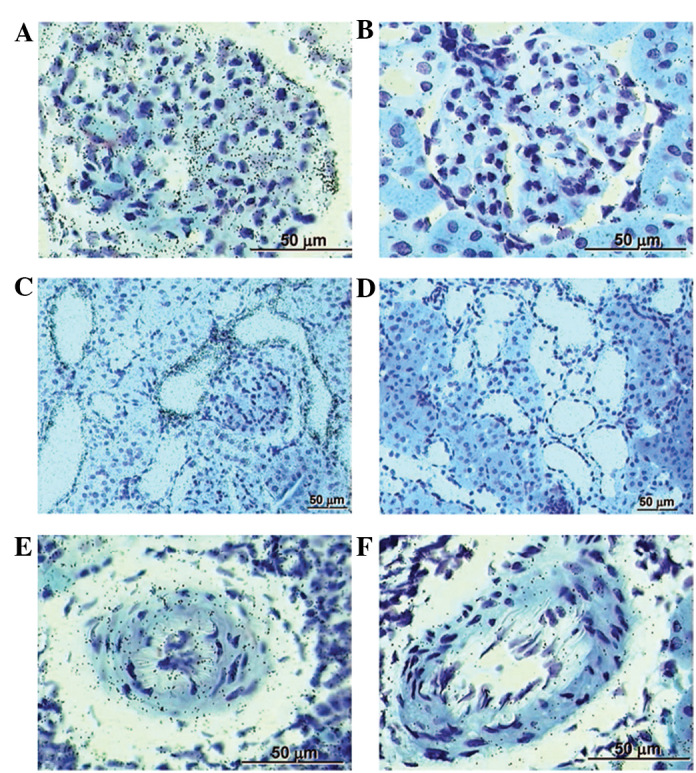
Saponin treatment suppresses the gene expression of the prorenin receptor (PRR) in spontaneously hypertensive rats (SHRs). (A) PRR in glomeruli of normal rats and (B) of SHRs (108 mg/kg per day); in tubules of (C) normal rats and of (D) SHRs (108 mg/kg per day); and (E) in small cortical vessels of normal rats and (F) of SHRs (108 mg/kg per day).

*In situ* hybridized renal sections from the placebo-treated controls showed prominent labeling for PRR in the glomeruli and tubules, with less labeling evident in the renal arteries (Fig. 4A, C and E). However, in the saponin-treated SHRs, the expression of PRR in these renal sections was significantly suppressed compared with the placebo-treated controls (Fig. 4B, D and F).

### Treatment with saponin increases renal rat renin gene expression

**Figure 5.**
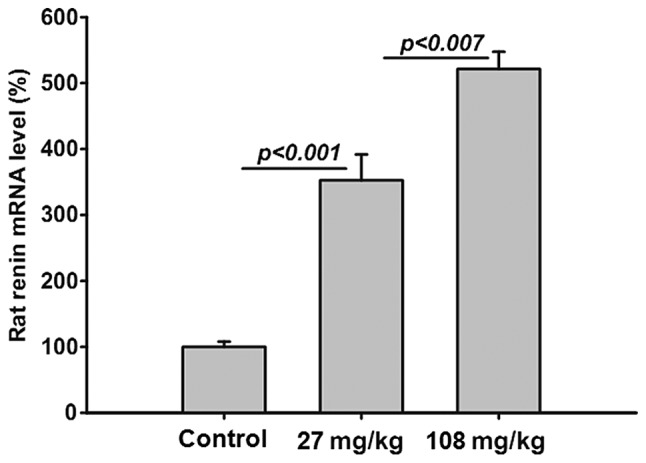
Saponin treatment increases renal rat renin gene expression in spontaneously hypertensive rats.

The gene expression of endogenous rat renin was significantly and dose-dependently increased in the saponin-treated SHRs (Fig. 5), indicating a saponin-induced RAAS blockade.

## Discussion

The aim of the present study was to investigate the protective effects of saponin on a hypertensive target organ (the kidney) in SHR rats as well as also to explore the effects of saponin on the RAAS.

Increased renin may lead to an increase in the BP of SHRs (9,10), thus, the observed inhibitory potency of saponin against rat renin suggests that saponin lowers BP in SHRs by inhibiting renin. The increased gene expression of renin in the renal samples of SHRs in the present study suggested an RAAS blockade.

The extensive level of saponin in the renal samples suggested a renoprotective effect via inhibition of the intra-renal RAAS. Moreover, autoradiographic grains observed in the glomeruli of each renal sample, which indicated the presence of saponin, suggested the potential for local renin inhibition in the glomeruli. Longer exposures to saponin may lead to the accumulation of saponin in other renal compartments. Notably, the presence of saponin in the vessel wall suggested that saponin may enter the granular cells of the afferent arteriole, the renin production site. Thus, it is possible that saponin may inhibit the production of renin prior to its release from the granular cells. A previous study has reported the blockade of intracellular renin by saponin in cultured myocardial cells (11).

In the present study, the development of albuminuria was prevented in the SHRs treated with saponin, but was not prevented in the placebo-treated controls. As albuminuria is considered a biomarker for the risk of renal decline these findings are relevant to the overall results (12). The anti-albuminuric effect of saponin was attributable to its anti-hypertensive effect.

TGFB1 in conjunction with Ang II plays a key role in renal fibrosis (13). The observations of the present study indicate that saponin suppressed the gene expression of TGFB1 in the renal samples of the SHRs allowing it to inhibit TGFB1-mediated pathways therefore leading them towards renal fibrosis. Saponin also reduced renal collagen I gene expression.

A possible correlation between the renoprotective effects of saponin and PRR has been explored in cardio-renal disease (14–16). The results of the *in situ* hybridization in the present study implicated saponin in the suppression of PRR gene expression *in vivo*. Incubation of saponin did not change the gene expression of PRR in the mesangial cells. The results showed a different distribution pattern of saponin in a greater number of renal compartments compared with that described previously in human kidneys (17).

In conclusion, the present study has demonstrated that saponin reduces systemic BP and blocks the circulating and tissue RAAS in SHRs. The administration of saponin was suspected to have a renoprotective effect via the inhibition of the intrarenal RAAS.
